# Increased high mobility group A 2 expression promotes transition of cervical intraepithelial neoplasm into cervical cancer

**DOI:** 10.18632/oncotarget.24080

**Published:** 2018-01-09

**Authors:** Liming Wang, Hui Shen, Da Zhu, Bei Feng, Lan Yu, Xun Tian, Ci Ren, Chun Gao, Xiaomin Li, Ding Ma, Zheng Hu, Hui Wang

**Affiliations:** ^1^ Key Laboratory of Cancer Invasion and Metastasis of the Ministry of Education, Tongji Hospital, Tongji Medical College, Huazhong University of Science and Technology, Wuhan, Hubei, China; ^2^ Department of Obstetrics and Gynecology, Tongji Hospital, Tongji Medical College, Huazhong University of Science and Technology, Wuhan, Hubei, China; ^3^ Department of Obstetrics and Gynecology, The First Affiliated Hospital, Sun Yat-Sen University, Guangzhou, Guangdong, China

**Keywords:** cervical cancer, cervical intraepithelial neoplasia (CIN), high mobility group A 2 (HMGA2), high-risk human papillomavirus (HR-HPV), apoptosis

## Abstract

Integration of the high risk human papillomavirus (HR-HPV) genome into host chromatin is an important step in cervical carcinogenesis. We identified HR-HPV integration sites within the human genome through detection of integrated papillomavirus sequences-PCR and assessed the role of high mobility group A 2 (HMGA2) in cervical carcinogenesis in clinical samples and cell lines. HPV integration sites were analyzed in 40 cervical cancer samples, while copy number variation and protein expression were assessed in 19 normal cervixes, 49 cervical intraepithelial neoplasia (CIN), and 52 cervical cancer samples. Overall, 25 HR-HPV integrating loci were detected in 24 cervical samples; *HMGA2* was the only recurring integration site. Both HPV copy number and HMGA2 protein expression were higher in cervical cancer than CIN samples. Area under the curve (AUC) values for HMGA2 expression and HPV copy number were 0.910 (95% CI: 0.844–0.976) and 0.848 (95% CI: 0.772–0.923), respectively. Expression of *Bcl-2* and *Caspase 3* can indicate the cell proliferation and apoptosis. Transfection of HMGA2 siRNA decreased HMGA2 mRNA and protein expression, *Bcl-2* expression, inhibited cell proliferation, and increased *Caspase 3* expression and apoptosis in SiHa, CaSki and S12 cervical cancer cells. HMGA2 overexpression had the opposite effects. These results suggest that elevated HMGA2 expression is associated with transformation of CIN into cervical cancer and that HMGA2 might be a useful biomarker for assessing the risk of cervical lesion progression.

## INTRODUCTION

Cervical cancer, the second most common cancer and the third leading cause of cancer-related death in women worldwide, creates substantial public health and financial burdens in developing countries such as China [1, 2]. Although high-risk human papillomavirus (HR-HPV) contributes to more than 90% of cervical cancer cases, HR-HPV infection is necessary but not sufficient for cancer development [3]. The *E6* and *E7* oncogenes of HR-HPV, which target the p53 and retinoblastoma (Rb) proteins, respectively, are crucial factors in cervical cancer carcinogenesis.

The high mobility group A 2 (HMGA2) protein belongs to a family containing 4 members: HMGA1a, HMGA1b, HMGA1c and HMGA2. The human *HMGA2* gene contains 5 exons located on the chromosome band 12q13-15 and spanning more than 140 kb. HMGA2 protein directly binds to DNA, modifying its conformation to allow binding of a group of transcriptional factors (TF). Also, the HMGA2 can inhibit tumor cell apoptosis by protecting the telomere [4]. Previous studies have identified *HMGA2* as a hot spot gene for HPV integration [5], and it can affect cell apoptosis by exerting opposite influences on *Bcl-2* and *Caspase 3* [6].

In this study, we used detection of integrated papillomavirus sequences-PCR (DIPS-PCR) to identify HPV integration loci in DNA obtained from cervical cancer tissues. We then examined *HMGA2* copy number variation in cervical cancer and CIN samples using fluorescence *in situ* hybridization (FISH). The HMGA2 levels in cervical cancer and CIN samples were also examined in an IHC assay. *Bcl-2* and *Caspase 3* expression were examined to evaluate the potential role of *HMGA2* in cervical carcinogenesis in SiHa, CaSki, and S12 cervical cancer cells. Real-time PCR (RT-PCR), western blot, a clone formation assay, and flow cytometry were used to determine the function of *HMGA2* and related genes in these cells.

## RESULTS

### Breakpoints in the human and HPV genomes

Integration of the HPV genome into the human genome was examined using DIPS-PCR. Integration loci were detected in 40 cervical cancer tissue samples. A total of 25 integration sites were identified in 24 cervical cancer samples; the remaining 16 samples were detected no integration. Most integrated HR-HPV sequences were characterized as HPV 16. The distribution of the integration breakpoints in the human and HPV genomes are shown in Table 1. Among the integration breakpoints identified, *HMGA2* was affected by HPV twice; the precise breakpoints were chr12-66095111 and chr12-66044325. These data suggest that *HMGA2* may be associated with cervical cancer.

**Table 1:** DIPS-PCR results

Sample	Chromosome	Breakpoint	Band	Gene	HPV	HPV breakpoint	HPV location
T-003	21	33534733	21q22.1	*HUNK*	16	1108	*E1*
T-007	1	153545227	1q21	*S100A2*	16	5518	*L2*
T-009	20	18215191	20p11.23	*PET117*	16	3863	*E5*
T-014	1	14479391	1p36.21	*PRDM2*	16	3077	*E2*
T-015	14	3616361	14q32.2	*BEGAIN*	16	6160	*L1*
T-016	12	66095111	12q13-15	*HMGA2*	16	1942	*E1*
T-018	1	107203056	1p13.3	*PRMT6*	16	3478	*E2, E4*
T-019	4	154004723	4q31.3	*FHDC1*	16	2187	*E1*
T-019	16	46389386	16q11.2	*SHCBP1*	16	4137	N.A.
T-021	11	93560735	11q21	*JRKL*	16	3016	*E2*
T-023	5	15518252	5p15.1	*FBXL7*	16	4199	N.A.
T-024	X	112171	Xp11.1-11.22	*UBQLN2*	16	5093	*L2*
T-025	12	66044325	12q13-15	*HMGA2*	16	3164	*E2*
T-026	2	133016766	2q21.2	*ZNF806*	16	4569	*L2*
T-028	5	125582273	5q23.2	*GRAMD3*	16	5077	*L2*
T-029	3	44021314	3q22-q23	*SOX14*	16	4742	*L2*
T-030	6	153288443	6q25.2	*FBXO5*	16	1922	*E1*
T-031	2	19758386	2p24.1	*OSR1*	16	2045	*E1*
T-032	5	1894297	5q33	*SOX30*	16	2891	*E2*
T-033	6	58281510	6p11.2	*GUSBP4*	16	1104	*E1*
T-034	10	60055745	10q21.1	*CISD1*	16	5092	*L2*
T-035	3	159389163	3q28	*IQCJ-SCHIP1*	16	2074	*E1*
T-036	6	25232282	6p22.3-p21.32	*FAM65B*	16	2544	*E1*
T-037	6	79774	6p22.2	*LRRC16A*	16	4837	*L2*
T-038	2	138417901	2q22.3	*ZEB2*	16	2901	*E2*

DIPS-PCR results for 40 cervical cancer samples. 25 integration positions were detected in 24 cervical cancer samples

HPV integration can cause DNA breakage and changes in gene expression, we conducted additional experiments focusing on *HMGA2*. FISH and IHC were used in CIN and cervical cancer tissue samples to examine *HMGA2* copy numbers and protein levels. We also assessed the potential of HMGA2 as a biomarker for progression from CIN to cervical cancer.

### *HMGA2* copy number variation and protein expression

Samples from 19 patients with normal cervical biopsies, 49 patients with CIN, and 52 patients with cervical cancer were examined in FISH and IHC assays. Cervical cancer stage was determined using International Federation of Gynecology and Obstetrics (FIGO) staging (Table 2).

**Table 2:** Patient data

	Normal	CIN			Cervical cancer	
		CINI	CINII	CINIII	CaI	CaII
**No. of cases**	19	49			52	
		17	16	16	29	23
**Age (years)**	48.74	46.80			44.38	
		48.41	45.88	46.00	45.10	43.48
**HPV copy number**	N.A.	0.99			2.05	
		0.92	0.94	1.12	1.80	2.37
***HMGA2* copy number**	1.91	1.81			1.83	
		1.68	1.99	1.77	1.78	1.89
***HMGA2* protein expression**	37882.30	16485.41			132507.00	
		28730.84	10146.52	9813.53	136380.20	127623.40

**Figure 1:**
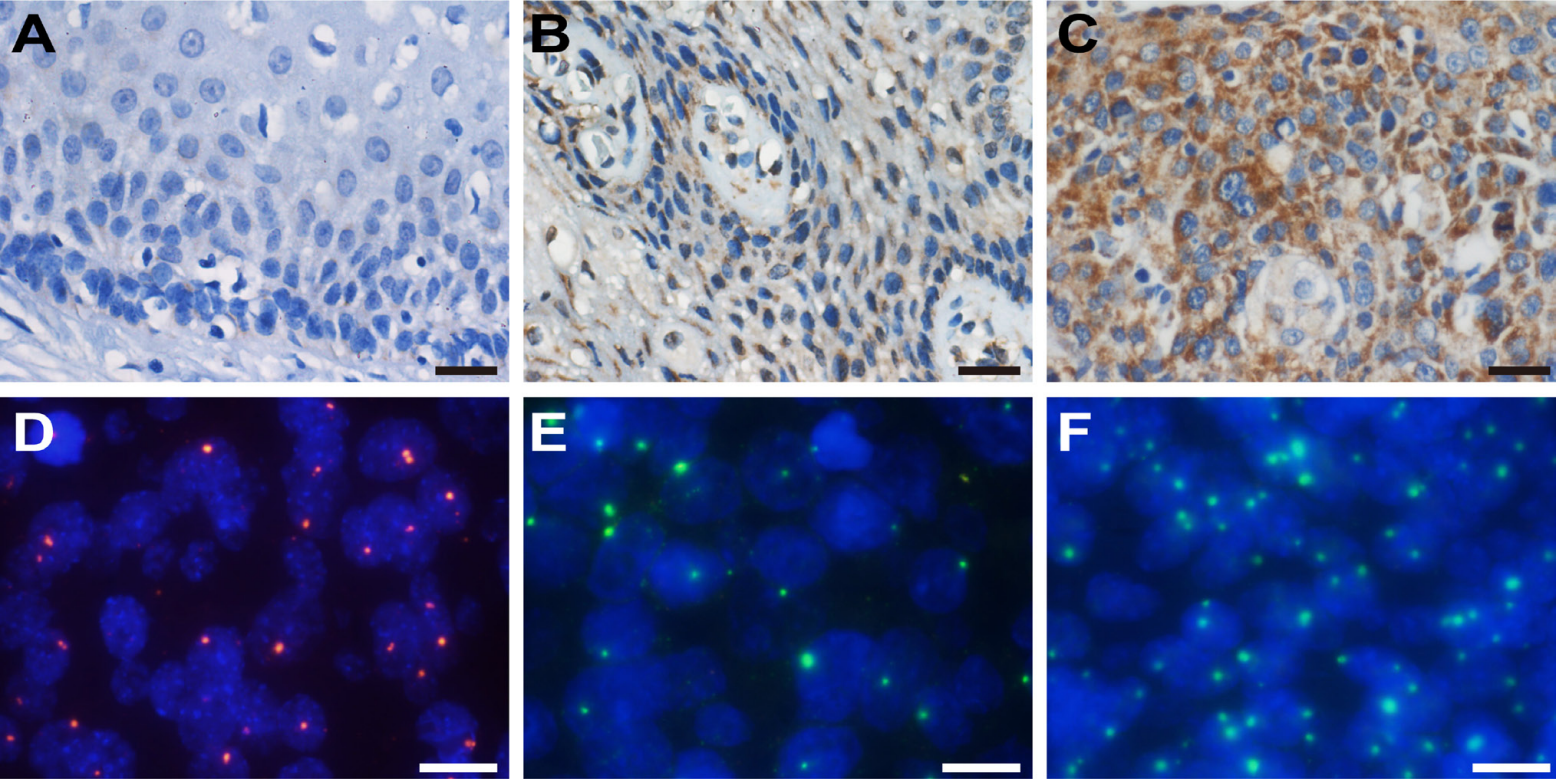
IHC staining of *HMGA2* and FISH detection of HPV and *HMGA2* Representative *HMGA2* staining in normal cervix (**A**), CIN (**B**) and cervical cancer samples (**C**) (×200). Scale bars, 20 μm. (**D**) HPV signals (red) in cervical lesions (×1000). (**E**) and (**F**) Variation in *HMGA2* signals (green) in different cervical lesions (×1000). Scale bars, 10 μm.

**Figure 2:**
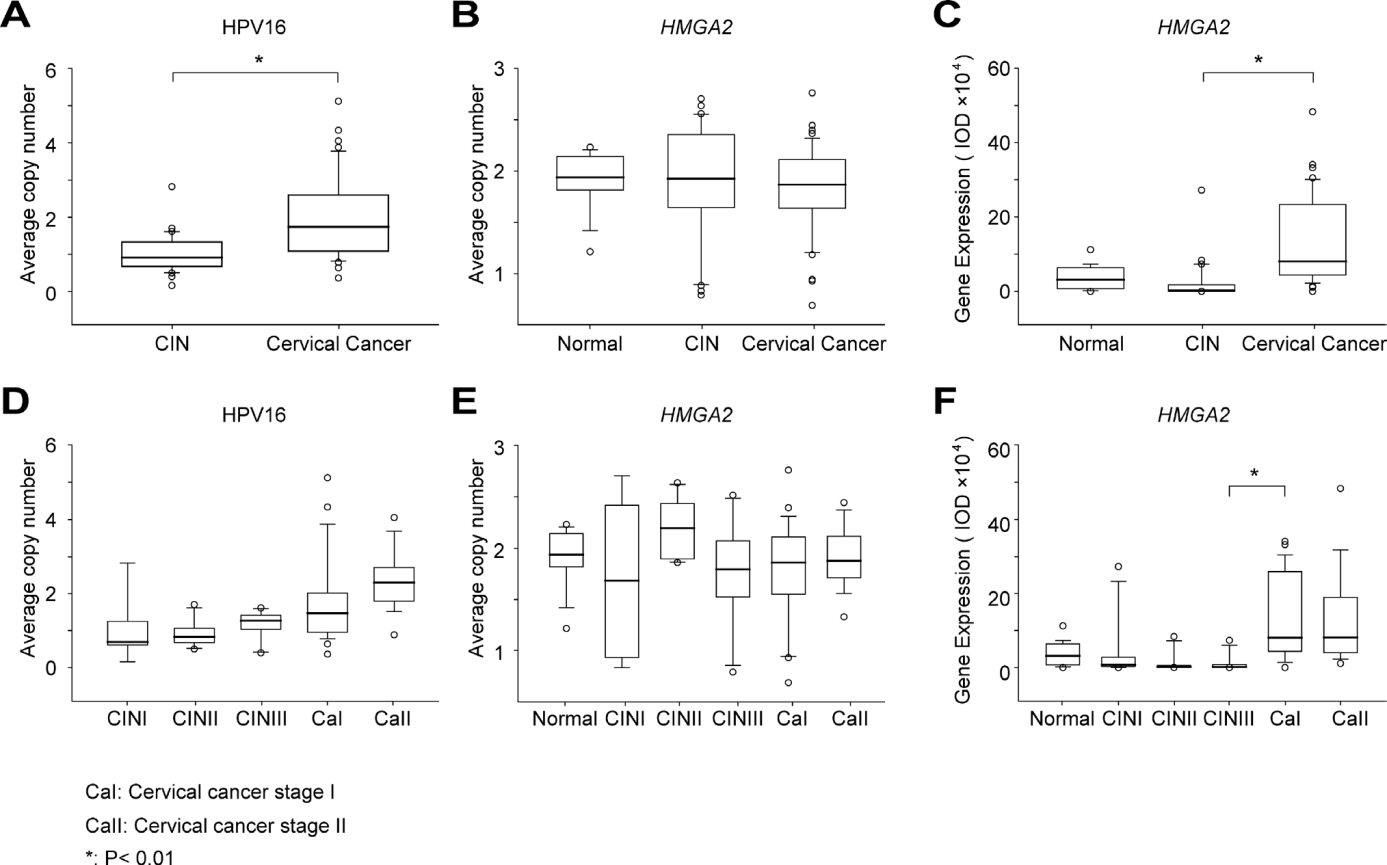
Variation in HPV copy number, *HMGA2* copy number, and HMGA2 protein expression Statistical analysis of HPV copy number (**A**), *HMGA2* copy number (**B**) and *HMGA2* protein expression (**C**) in normal cervical biopsies, CIN and cervical cancer samples. HPV copy number (**D**), *HMGA2* copy number (**E**) and HMGA2 protein expression (**F**) in cervical lesions of different stages. The original data are shown in Supplementary Table 3.

Variation in *HMGA2* and HPV copy numbers among normal, CIN, and cervical cancer tissue samples were examined using FISH. However, HPV signals were not detected in any of the normal cervical biopsy samples. Typical FISH signal patterns are shown in Figure 1. HPV copy numbers were higher in cervical cancer samples than in CIN samples (Figure 2A and Table 2, 2.05 vs. 0.99, *P* < 0.01). However, there were no significant differences in HPV copy number between any of the successive cervical lesions (Figure 2D; CINI vs. CINII, *P* = 0.938; CINII vs. CINIII, *P* = 0.136; CINIII vs. stage I cervical cancer; *P* = 0.043; stage I vs. stage II cervical cancer, *P* = 0.052).

*HMGA2* copy numbers did not differ between normal cervical biopsy samples and either CIN or cervical cancer samples (*P* = 0.499 and *P* = 0.457, respectively) (Figure 2B). Furthermore, there were no significant differences in *HMGA2* copy number between any of the successive CIN and cervical cancer stages (Figure 2E; CINI vs. CINII, *P* = 0.121; CINII vs. CINIII, *P* = 0.163; CINIII vs. stage I cervical cancer; *P* = 0.948; stage I vs. stage II cervical cancer, *P* = 0.288).

**Figure 3:**
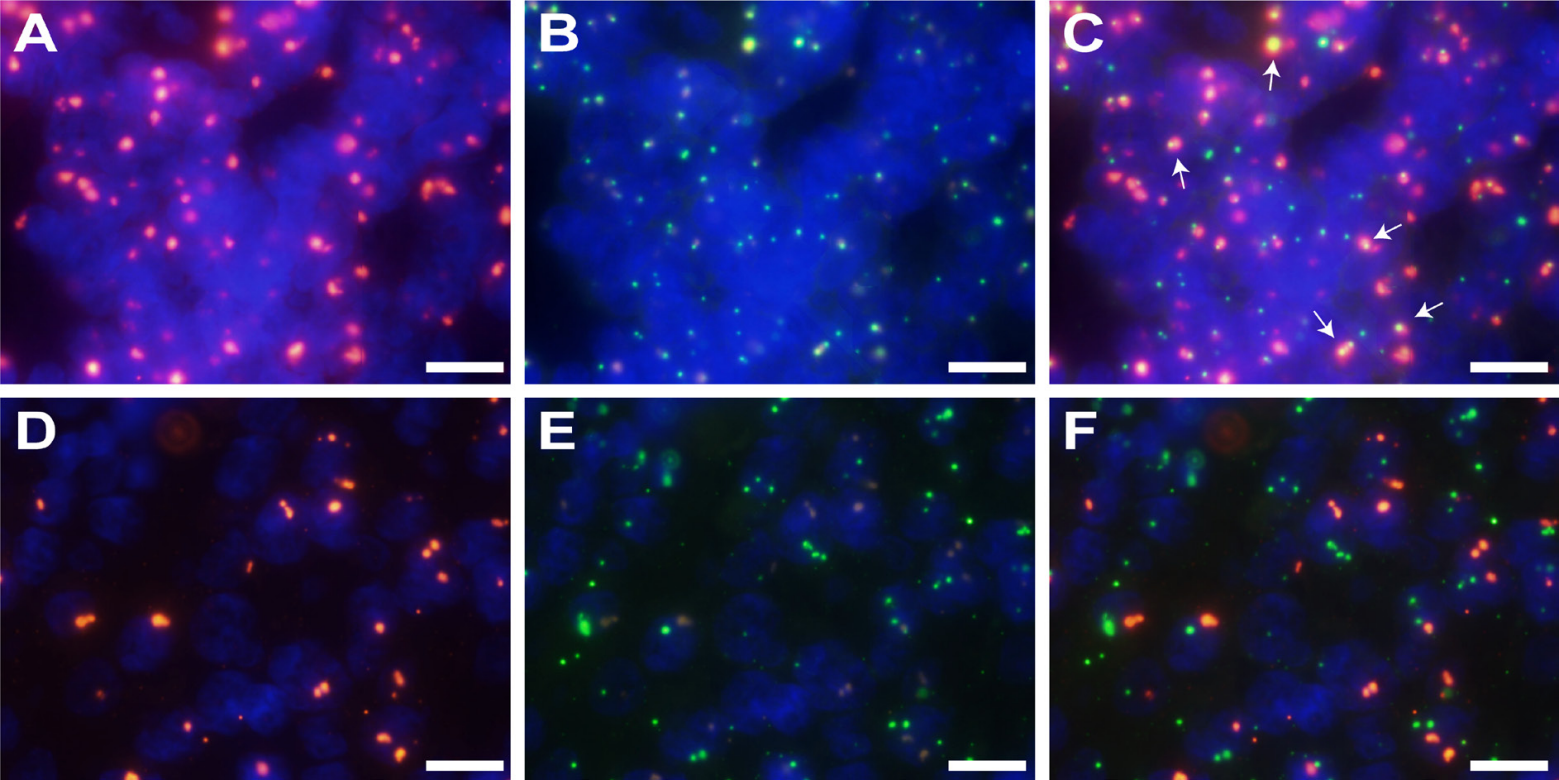
FISH signals showing integration of HPV into the *HMGA2* gene (**A**–**C**) Fusion signals for HPV and *HMGA2* (×1000). (A) HPV signal (red) in sample T-025; (B) *HMGA2* signal (green) in sample T-025; (C) Fusion image of (A) and (B). Arrows shows merged signals (yellow). (**D**–**F**) Samples without HPV integration showed no fusion signals (×1000). (D) HPV signal (red) in sample T-011; (E) *HMGA2* signal (green) in sample T-011; (F) Fusion image of (D) and (E). Scale bars, 10 μm.

HPV (shown in red) and *HMGA2* (shown in green) copy numbers were also detected in the same samples by FISH. Fusion signals were detected in each of the two samples for which DIPS-PCR indicated that HPV was integrated into the *HMGA2* gene. Fusion signals were absent in all other samples in which HPV was not integrated into *HMGA2* (Figure 3).

Representative HMGA2 IHC staining patterns are shown in Figure 1. Average IOD values were much higher in cervical cancer samples than in CIN samples (Figure 2C and Table 2, 132507.00 vs. 16485.41, *P* < 0.01). *HMGA2* IOD values were also much higher in stage I cervical cancer than in CINIII samples (Figure 2F and Table 2, 136380.20 vs. 9813.53, *P* < 0.01); no other significant differences were observed between successive stages of CIN and cervical cancer (Figure 2F; normal vs. CINI, *P* = 0.589; CINI vs. CINII, *P* = 0.286; CINII vs. CINIII, *P* = 0.962; stage I vs. stage II cervical cancer, *P* = 0.791).

**Figure 4:**
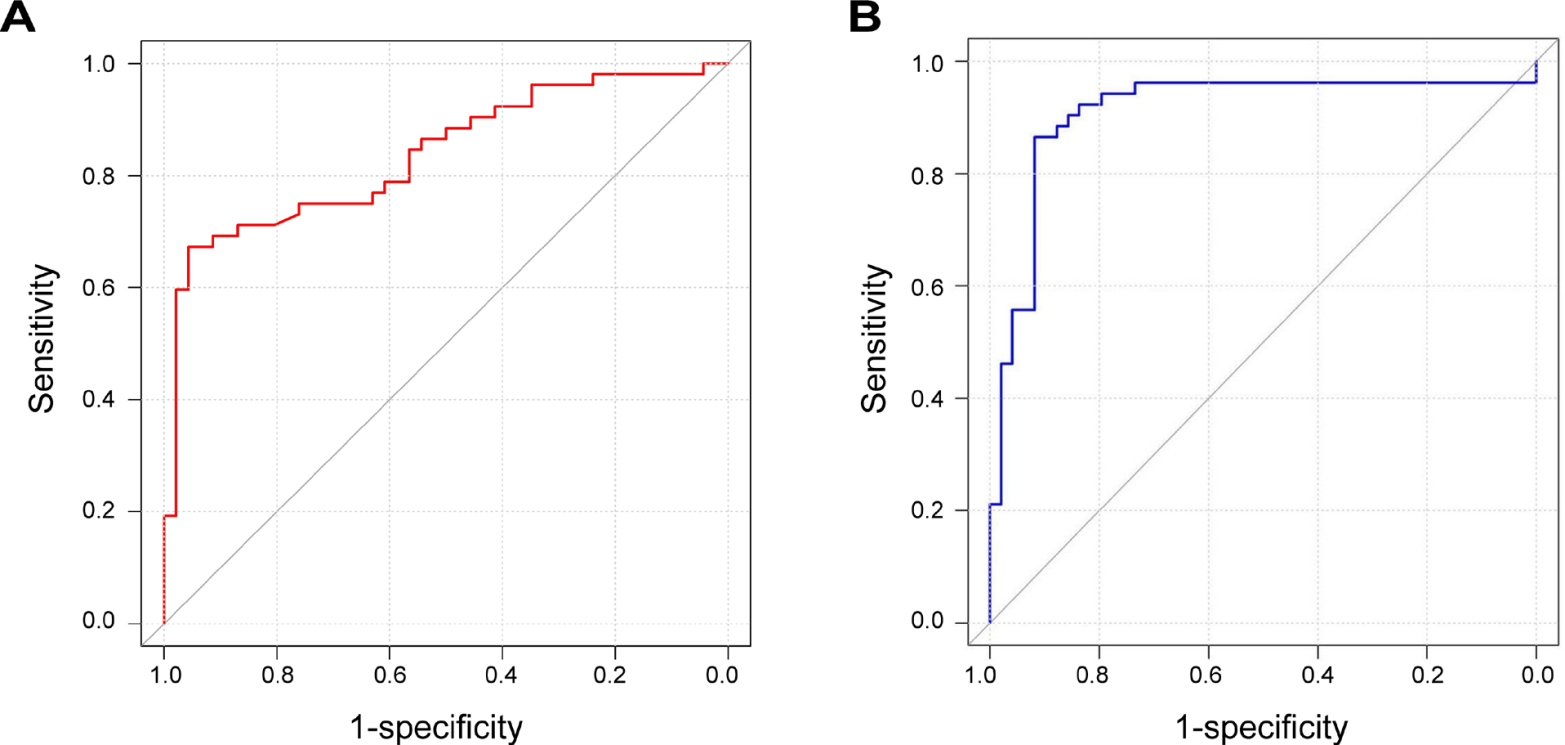
ROC curves for HPV copy number and *HMGA2* protein expression (**A**) ROC curve for HPV copy number. The AUC is 0.848 with a 95% CI of 0.772–0.923. (**B**) ROC curve for *HMGA2* protein expression. The AUC is 0.910 with a 95% CI of 0.844–0.976.

HPV copy number and *HMGA2* protein expression were evaluated as biomarkers for the transition of CIN into cervical cancer using receiver operating characteristic (ROC) curve analysis. Maximum values of Youden's index were used as cutoff values. The area under the curve (AUC) for HPV copy number was 0.848 (95% CI: 0.772–0.923), and the cutoff value was set at 1.58. The sensitivity and specificity of HPV copy number were 67.3% and 95.7%, respectively. For HMGA2 expression, the AUC was 0.910 (95% CI: 0.844–0.976), the cutoff value was 29949.61, the sensitivity was 86.5%, and the specificity was 91.8% (Figure 4).

**Figure 5:**
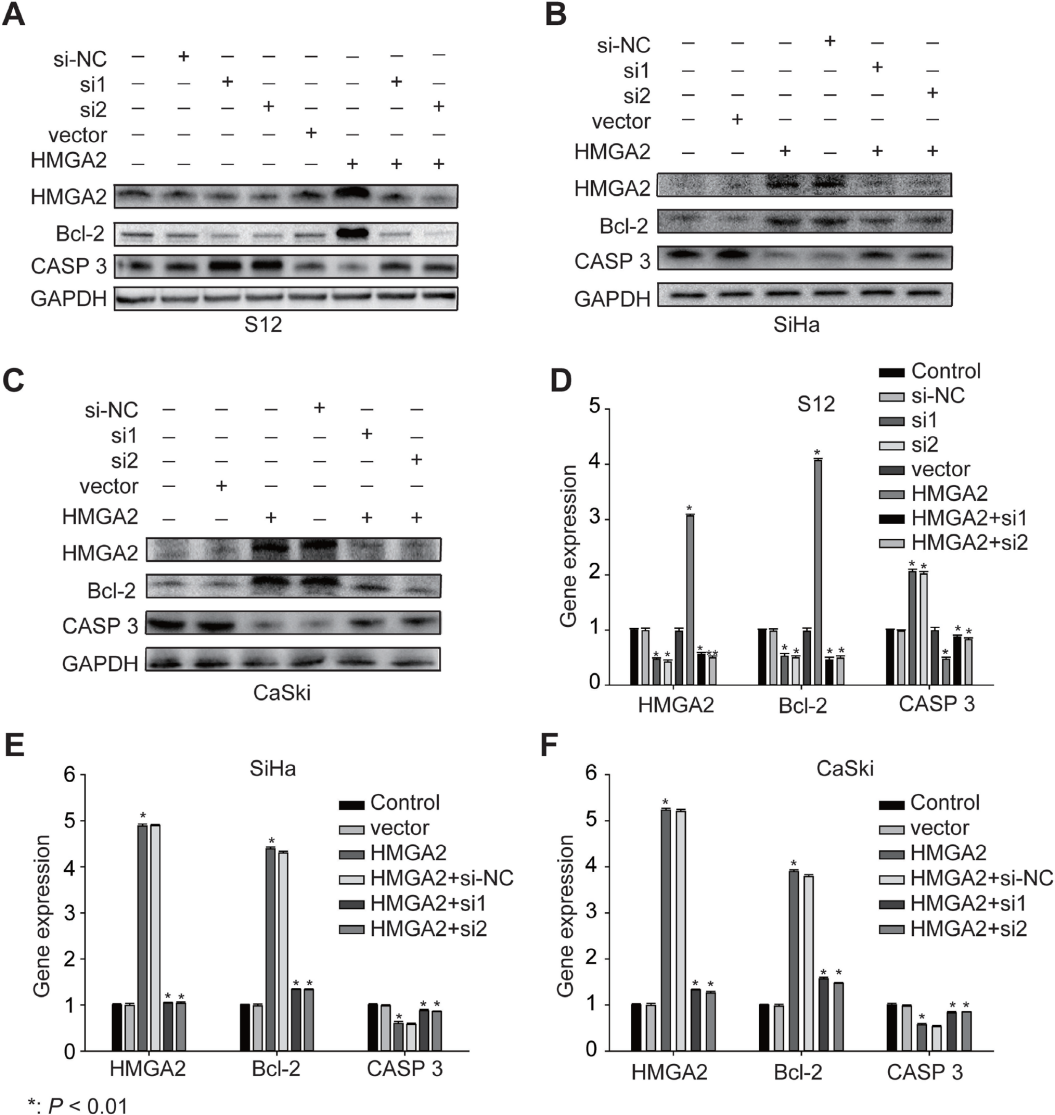
Expression of *HMGA2* and its related genes in S12, SiHa, and CaSki cells Western blot analysis of *HMGA2, Bcl-2*, and *Caspase 3* protein expression in S12 (**A**), SiHa (**B**), and CaSki (**C**) cells and control cells after treatment with *HMGA2* overexpression plasmid or siRNAs. Real-time PCR assay of *HMGA2, Bcl-2*, and *Caspase 3* in expression in S12 (**D**), SiHa (**E**), and CaSki (**F**) cells and control cells after treatment with *HMGA2* overexpression plasmid or siRNAs. The data represent the mean ± SD (*n* = 3). ^*^P < 0.01.

SiHa, CaSki, and S12 cell lines were used to explore the role of *HMGA2* in cervical carcinogenesis. HMGA2 expression is higher in S12 cells, which have HPV 16 integration locus in *HMGA2* [5], than in SiHa and CaSki cells. Transfection of the *HMGA2* over-expression plasmid increased HMGA2 RNA and protein levels, up-regulated *Bcl-2* expression, and down-regulated *Caspase 3* expression in S12. In contrast, HMGA2-siRNA decreased HMGA2 expression, in turn increasing Caspase 3 and decreasing *Bcl-2* mRNA and protein expression (Figure 5). We first up-regulated *HMGA2* by *HMGA2* over-expression plasmid in SiHa and CaSki, and then the cells were incubated with HMGA2-siRNAs. We found the expression of Bcl-2 was decreased and the expression of *Caspase 3* was increased in RNA and protein levels (Figure 5).

**Figure 6:**
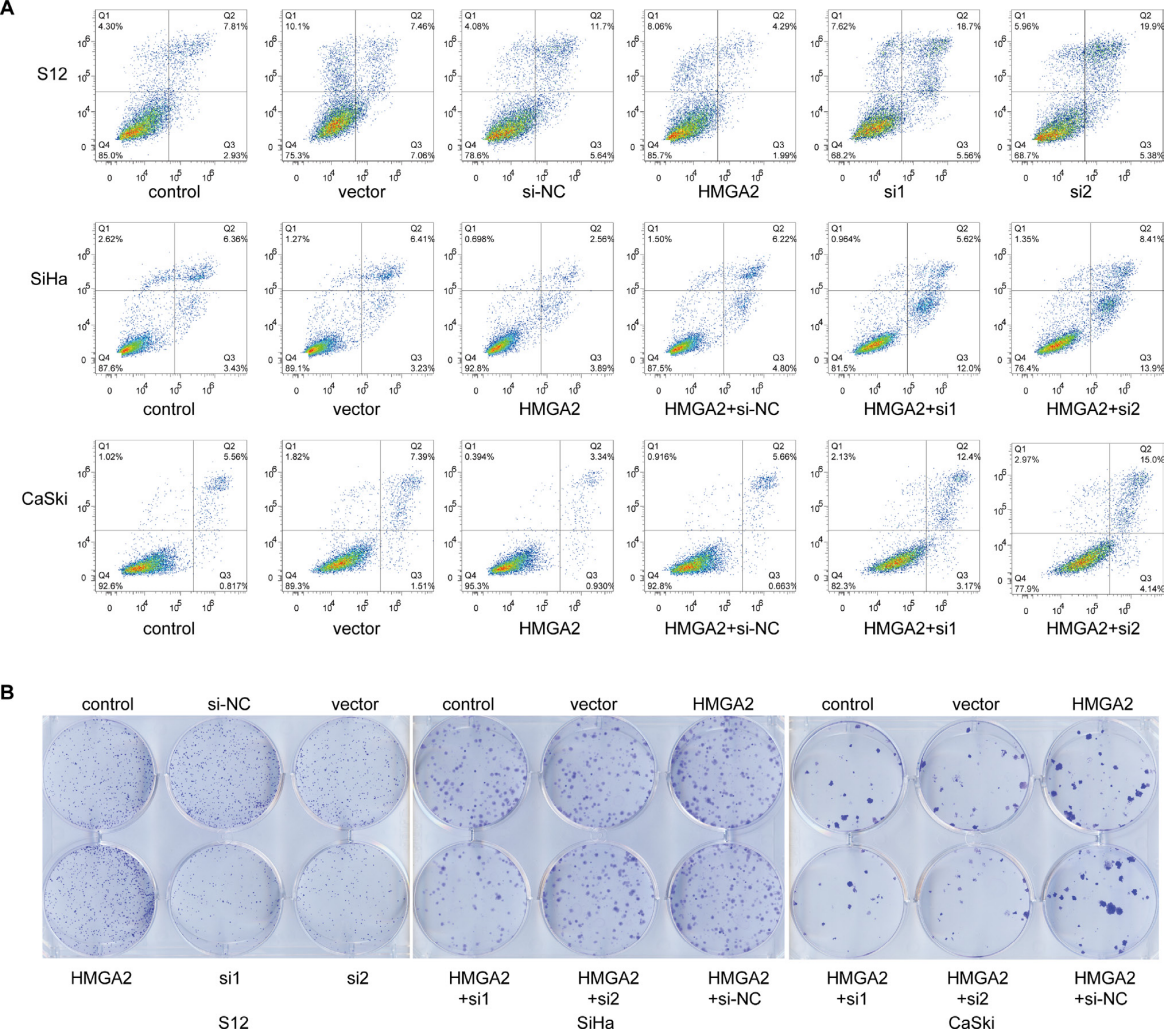
*HMGA2* inhibited cell apoptosis and increased proliferation (**A**) Representative cell apoptosis images of S12, SiHa, and CaSki cells 48 hours after transfection with HMGA2 overexpression plasmid or siRNAs were obtained using flow-cytometry. (**B**) Representative cell clone formation images of S12, SiHa, and CaSki cells 2 weeks after transfection with HMGA2 overexpression plasmid or siRNAs.

In addition, we detected the cell apoptosis by flow cytometry. Compared with the apoptosis rate of control group and *HMGA2* vector group (10.74% and 14.52%, respectively), the apoptosis rate transfected with *HMGA2* over-expression plasmid was reduced in S12 cells (6.28%). Furthermore, the apoptosis rates transfected with HMGA2-siRNAs were 24.26% and 25.28%, which were higher than siRNA control group (17.34%) (Figure 6A). SiHa and CaSki cells transfected with *HMGA2* over-expression plasmid and then with HMGA2-siRNAs showed higher apoptosis (17.62% and 22.31% in SiHa, 15.57% and 19.14% in CaSki) than co-transfected with *HMGA2* over-expression plasmid and siRNA control (11.02% in SiHa, 6.32% in CaSki) (Figure 6A). All cell lines with elevated *HMGA2* expressions also tended to have increased cell proliferation, while cells in which HMGA2 expression was inhibited tended to have and decreased cell proliferation (Figure 6B).

## DISCUSSION

Recent studies have demonstrated that genomic instability and HPV integration contribute to cervical cancer tumorigenesis. Integration of HPV into certain parts of the genome can activate proto-oncogenes or inhibit tumor suppressor gene expression; for example, integration of HPV 18 into a region close to the 8q24 locus led to persistent expression of the oncogene *c-MYC* in the HeLa cell line [7]. Alterations like these could promote cervical cancer carcinogenesis. Previous studies have also shown that HPV integration causes DNA damage and repair, thereby leading to genomic instability [8]. The presence of gene deletions and insertions near HPV integration loci in cervical cancer cell lines and clinical samples suggests that genome instability caused by HPV integration, which can disrupt gene expression, may be a hallmark of cervical cancer [5].

Here, we used DIPS-PCR to identify HPV integration sites in the human genome. The *HMGA2* gene was identified as one such locus. HMGA2 is a non-histone chromatin protein encoded by the *HMGA2* gene located in region 12q13-15. HMGA2 protein, which binds to the minor groove of AT-rich DNA sequences to alter chromatin structure and regulates the transcription of several genes, is expressed widely during embryogenesis but at low levels in adult tissues [9]. Chromosomal rearrangements involving the region around the *HMGA2* gene have been reported in several benign mesenchymal tumors [10]. In malignant tumors, *HMGA2* overexpression has been reported in esophageal [11] and lung [12] cancers. Elevated HMGA2 expression is associated with a highly malignant phenotype, poor prognosis, increased metastasis, and reduced survival [13, 14]. As a DNA binding protein, HMGA2 regulates the cell cycle by binding to the crucial regulator pRb [15] and inducing cyclin A expression [16]. These findings suggest that *HMGA2* may also play an important role in cervical cancer. We therefore investigated the utility of HMGA2 as a biomarker for cervical cancer by assessing genomic instability of the *HMGA2* locus using FISH and *HMGA2* protein expression using IHC.

We found that HPV copy number and *HMGA2* protein expression both increased as CIN progressed into cervical cancer, but *HMGA2* copy number did not. This indicates that, unlike *c-MYC* in the 8q24 location, there was no obvious chromosomal rearrangement in the 12q13-15 region during cervical cancer tumorigenesis. HPV signals were not detected in the normal cervical biopsy samples. However, in accordance with the DIPS results, HPV and *HMGA2* fusion signals were detected in two clinical samples. HMGA2 expression increased dramatically from CINIII to stage I cervical cancer, suggesting that HMGA2 is an important factor in the progression of CIN into cervical cancer. HPV copy numbers also increased as CIN progressed into cervical cancer, suggesting that HPV is more extensively integrated into the genomes of cervical cancer cells than of CIN cells. We then examined the diagnostic value of HPV copy number and HMGA2 expression using ROC curves; AUC values are indicative of utility as a diagnostic biomarker. HMGA2 expression, with an AUC value of 0.910 (95% CI: 0.844–0.976), was a better potential diagnostic biomarker for distinguishing between cervical cancer and CINs than HPV copy number, which had an AUC value of 0.848 (95% CI: 0.772–0.923). The cutoff value determined using Youden's index and high sensitivity and specificity values indicate that HMGA2 levels increase dramatically when CIN progresses into cervical cancer. This suggests HMGA2 may play a crucial role in the transition of CIN into cervical cancer.

Because the proto-oncogene *HMGA2* may play a crucial role in the transition of CIN into cervical cancer, treatments targeting HMGA2 might be potential therapeutic strategies. In this study, integration of HPV into the *HMGA2* gene was detected in only 2 samples, but HMGA2 protein expression was elevated in a much larger proportion of the cervical cancer samples. This indicates that HPV integration can at most account for only part of the observed overexpression of HMGA2 in cervical cancer. We also examined the mechanisms by which HMGA2 might contribute to cervical carcinogenesis in SiHa, CaSki, and S12 cells. Bcl-2 (B-cell lymphoma 2) is a member of the Bcl-2 family and regulates cell death (apoptosis) by either inducing (pro-apoptotic) or inhibiting (anti-apoptotic) apoptosis [17, 18]. Caspases (cysteine-aspartic proteases) are a family of protease enzymes playing essential roles in programmed cell death (including apoptosis) and inflammation. *Caspase 3* is a member of the caspase family, and over-activation of caspase-3 can lead to excessive programmed cell death [19]. We chose *Bcl-2* and *Caspase 3* to demonstrate the potential role of *HMGA2* in cervical carcinogenesis. Down-regulation of *HMGA2* reduced *Bcl-2* expression and increased Caspase 3 expression (Figure 5), which in turn reduced cell proliferation and increased apoptosis rates (Figure 6), in all three cell lines. In contrast, up-regulation of HMGA2 increased Bcl-2 expression, decreased Caspase 3 expression, increased cell proliferation, and decreased apoptosis rates (Figures 5 and 6). These data suggest that HMGA2 promotes cell proliferation and tumor malignancy.

## MATERIALS AND METHODS

### Tissue samples and data collection

A total of 49 CIN and 52 cervical cancer patients were recruited at Tongji Hospital between 2014 and 2017. As negative control, 19 patients from the Gynecological Department who underwent hysterectomies and were confirmed to have normal cervixes were also recruited. The samples were collected via surgery and biopsy. Pathological diagnoses were evaluated and confirmed by two different pathologists from the Pathology Department of Tongji Hospital. All patients provided written informed consent. All experiments were approved by the ethical committee of Tongji Hospital. DNA was extracted from 40 fresh cervical cancer tissue samples; all normal cervix, CIN, and cervical cancer tissue samples were formaldehyde-fixed and paraffin-embedded, and slides with 4-μm thick sections of these samples were obtained. One slide per sample was stained with hematoxylin and eosin and examined by a pathologist to confirm CIN or cervical cancer. Clinical data were obtained from individual patient charts. International Federation of Gynecology and Obstetrics (FIGO) staging was used.

### DNA extraction from clinical tissue samples

Genomic DNA was extracted from clinical samples using a Tissue DNA Kit (D3396-01 E.Z.N.A., Doraville, GA, USA). Genomic DNA was dissolved in triple-distilled water and concentration was determined using a Nanodrop 2000 spectrophotometer (Thermo Fisher ND 2000); all DNA samples were stored at −80°C.

### Detection of integrated papillomavirus sequences-PCR (DIPS-PCR)

Detection of integrated papillomavirus sequences via ligation mediated PCR (DIPS-PCR) was performed to identify HPV integration loci in sample DNA. The assay was performed as described by Luft *et al.* [20]. Briefly, the Sau3AI-specific ds-adapter was ligated to genomic DNA after it had been digested with Sau3AI. First-round linear and second-round exponential PCR were then conducted. Primers used in this procedure are listed in Supplementary Table 1. Products of exponential PCR were analyzed on 2% agarose gels, stained with Cyber Green, and measured using UV light on an ChemiImager 5500 (Alpha Innotech, Kasendorf, Germany). All products were sequenced at Tianyi Biotechnology, Ltd.

### Analysis of PCR product sequences

Sequencing results were analyzed using NCBI nucleotide blast (http://blast.ncbi.nlm.nih.gov) with the hg19 assembly.

### Fluorescence *in situ* hybridization (FISH)

A 1899 bp-long sequence of human *HMGA2* CDS (NCBI Reference Sequence: NM_003483.4) was synthesized and ligated to the PUC57 vector as a template. This CDS sequence was amplified with the following primers: forward, 5′-GGAGTCTCCCCATCCTCCTT-3′; reverse, 5′-TCGCTCCTCCCACCTCATAA-3′. The complete sequence is shown in Supplementary Table 2. The whole genome plasmid for HPV type 16 (HPV-16) was a kind gift from Harald zur Hausen. The probes were labeled using standard nick translation with digoxigenin-dUTPs or biotin-dUTPs. The slides were dewaxed, rehydrated, pre-treated with hydrogen peroxide, dehydrated again, digested, and dehydrated once more. The probe and target DNA were denatured simultaneously prior to hybridization overnight. After hybridization, the preparations were stringently washed with 2× SSC containing formamide. The probe was detected using peroxidase-conjugated sheep anti-digoxigenin Fab fragments (1:200; Roche), Cy3-conjugated streptavidin (1:100; Roche), or FITC-labeled tyramide (1:50, Perkin Elmer) and mounted with Vectashield (Vector Laboratories) containing 4′,6-diamidino-2-phenylindole (DAPI). The results were analyzed under a fluorescence microscope (Olympus BX53) using Cellsens Fluorescence imaging system software. Copy numbers were evaluated based on numbers of punctate signals per nucleus.

### Immunohistochemistry (IHC)

IHC was used to examine HMGA2 protein expression. IHC reactions were performed on slides following a standard 3,3′-Diaminobenzidine (DAB) staining protocol. Briefly, the slides were dewaxed, rehydrated, washed with water, and then pre-treated with 3% hydrogen peroxide. The slides were boiled in antigen retrieval solution, blocked with 1% BSA, and incubated overnight in a humidified chamber at 4°C with an anti-HMGA2 monoclonal antibody (1:50, Abcam, ab97276). After incubations with biotinylated antibody and streptavidin-peroxidase, staining was developed in a solution of 3,3′-Diaminobenzidine. Slides were then counterstained with hematoxylin, dehydrated, cleared with xylene, and mounted. Image pro plus (IPP) software was used to analyze the staining. Integral optical density (IOD) of staining was analyzed.

### Cervical cancer cell lines, plasmids, small interfering RNA (siRNA) and transfection

The SiHa and CaSki cervical cancer cell lines were purchased from ATCC, and the S12 cell line, an immortalized human cervical keratinocyte cell line, was a kind gift from Prof. Kenneth Raj (Health Protection Agency) with the permission of the original owner, Prof. Margaret Stanley [21]. SiHa and CaSki cells were cultured in Dulbecco's Modified Eagle Medium (DMEM) supplemented with 10% fetal bovine serum (FBS, Gibco) and 100 U penicillin and streptomycin (Invitrogen). S12 cells were maintained in a 1:3 mixture of DMEM and Ham's F12 (Gibco) medium supplemented with 5% FBS, 24.3 mg/mL adenine, 0.5 mg/mL hydrocortisone, 5 mg/mL insulin, 10 ng/mL epidermal growth factor, and 8.4 ng/mL cholera toxin. All cell lines were incubated at 37°C in humidified incubator with 5% CO_2_. The *HMGA2* overexpression plasmid and its vector were purchased from Vigene Bioscience Inc. (Shandong, China). siRNAs for HMGA2 with the following sequences were purchased from Suzhou Ribo Life Science Co., Ltd.: siRNA 1, sense 5′-CCUCUAAAGCAGCUCAAAA-3′, anti-sense 5′-GG AGAUUUCGUCGAGUUUU-3′; siRNA 2, sense 5′-CA AGAGGCAGACCUAGGAA-3′, anti-sense 5′-GUUCUC CGUCUGGAUCCUU-3′. All the cells were transfected using Lipofectamine 3000 (Thermo Fisher Scientific) according to the manufacturer's instructions. Experiments were performed three times in duplicate.

### Real-time PCR

Total RNA was extracted from cells with the RNeasy Mini Kit (74104, Qiagen, Germany). The extracted mRNA was reverse transcribed into single-stranded complementary DNA (cDNA) using Reverse Transcriptase M-MLV (2641A, Takara, Japan). For quantitative real-time PCR for *HMGA2*, *Bcl-2*, and *Caspase 3*, amplification mixtures were prepared using iTaq™ Universal SYBR Green supermix (172–5124, Bio-Rad, USA). Primers were designed using PrimerBank (https://pga.mgh.harvard.edu/primerbank/). GAPDH was used as an internal reference gene to normalize expression values. Results were expressed as ratios of reference to target gene expression using the 2−ΔΔCt method. Statistical analyses were performed with GraphPad Prism 5. Primers used for RT-PCR are shown in Supplementary Table 4.

### Western blot analysis

Cells were lysed for 30 minutes in ice-cold RIPA lysis buffer (Servicebio Inc, Wuhan, China) and a protease inhibitor cocktail (Roche). The following primary antibodies used: rabbit anti-GAPDH (1:2000, Antgene, Wuhan, China), rabbit anti-*HMGA2* (1:200, A12743, ABclonal), rabbit anti-Bcl-2 (1:1000, 12789-1-AP, Proteintech), and rabbit anti-Caspase 3 (1:1000, A11953, ABclonal). Protein was detected with horseradish peroxidase-conjugated anti-rabbit IG secondary antibody using the ECL system (Bio-Rad).

### Flow cytometry apoptosis assay

Forty-eight hours after transfection, cells were collected and double-stained with fluorescein isothiocyanate- (FITC-) conjugated annexin V (annexin V-FITC) and propidium iodide (PI) using an Annexin V-FITC Apoptosis detection kit (556547, BD Bioscience) according to the manufacturer's instructions. Apoptosis rates for all cell lines were measured using a FACS Caliber (BD Bioscience). Data was analyzed using FlowJo software.

### Clone formation assay

A total of 200 cells were plated in triplicate in DMEM medium with 10% FBS in 6-well plates and incubated for 2 weeks. Clones were stained with 0.04% crystal violet and photographed.

### Statistical analysis

Copy numbers and protein expression were compared using Student's *t*-tests and analysis of variance. *P* < 0.01 was considered having statistically significant difference. A receiver operating characteristic (ROC) curve analysis was used to evaluate the diagnostic capabilities of HPV copy number and *HMGA2* copy number and protein expression. The maximum values of Youden's index were used as the optimum cutoff values in the evaluation of these potential diagnostic biomarkers.

## SUPPLEMENTARY MATERIALS TABLES




