# Supplementary material for: Increased high mobility group A 2 expression promotes transition of cervical intraepithelial neoplasm into cervical cancer
Source: Oncotarget. 2018 Jan 9;9(8):7891–901. doi: 10.18632/oncotarget.24080 (PMC5814267; doi:10.18632/oncotarget.24080)
Supplement: Supplementary file 1 [file oncotarget-09-7891-s001.pdf]

Increased high mobility group A 2 expression promotes transition of cervical intraepithelial neoplasm into cervical cancer

SUPPLEMENTARY MATERIALS

Supplementary Table 1: DIPS-PCR primers of HPV16, HPV18 and primers used in the formation of Sau3AI-specific adapters

HPV16-1 first round	GGGATGTAATGGATGGTTTTATGT
HPV16-2 first round	GGCGCCATGAGACTGAAACACC
HPV16-3 first round	GCCAGAATGGATACAAAGACAAACA
HPV16-4 first round	TTTGCACGAGGACGAGGACA
HPV16-5 first round	AGAGCCAGACACCGGAAACC
HPV16-rev-1 first round	ACACAACAAACAACACTAATTCAACAT
HPV16-rev-2 first round	GCACCAAAGCCAGTATGAACC
HPV16-rev-3 first round	AGCACCTATAGATTTTCCACTACGAG
HPV16-rev-4 first round	AAGTTGGGTAGCCGATGCAC
HPV16-1 second round	GGGATGCTATATCAGATGACGAGAACG
HPV16-2 second round	GTGGAAGTGGGGGTGGTTGC
HPV16-3 second round	GGTACAATGGGCCTACGATAATGA
HPV16-4 second round	ACGAGGACAAGGAAAACGATGGAGA
HPV16-5 second round	GAAACCCCTGCCACACCACT
HPV16-rev-1 second round	TGAGGTGGTGGGTGTAGCTTT
HPV16-rev-2 second round	AATGGTGGACAATCACCTGGA
HPV16-rev-3 second round	AATGCCAGTACGCCTAGAGGTT
HPV16-rev-4 second round	CAGAACGTTTGTGTCGCATTG
HPV18-1 first round	CAGAAGGTACAGACGGGGAGG
HPV18-2 first round	TAGACAACGGGGGCACAGAGG
HPV18-3 first round	CACCAAAATTGCGAAGTAGTGT
HPV18-4 first round	AGGAAGAGGAAGATGCAGACACC
HPV18-5 first round	CCTACAGGCAACAACAAAAGACG
HPV18-rev-1 first round	TCCAGTATCTACCATATCACCATCTTCC
HPV18-rev-2 first round	TAGTGTCCACAGGCTCAAAGG
HPV18-rev-3 first round	TGGAAATAGACACAGAGGTAGACGAAG
HPV18-rev-4 first round	AGGGGACGTTATTACCACAATATACAC
HPV18-1 second round	GGGTTGTAACGGCTGGTTTTATGT
HPV18-2 second round	GGGCACAGAGGGCAACAACA
HPV18-3 second round	AATGGGAGACACACCTGAGTGGATACA
HPV18-4 second round	AGATGCAGACACCGAAGGAAACCC
HPV18-5 second round	AGGCAACAACAAAAGACGGAAACT
HPV18-rev-1 second round	ATCTACCATATCACCATCTTCCAAAACT
HPV18-rev-2 second round	GAGGATGGACGTGTAAGAAACTCAGG
HPV18-rev-3 second round	ATCAAACCCAGACGTGCCAGTAAA
HPV18-rev-4 second round	TACAGACAGATGGCAAAAGCGG
AL1	GGGCCATCAGTCAGCAGTCGTAGCCGGATCCAGACTTACACGTTG
AP1	GGCCATCAGTCAGCAGTCGTAG
AS-Sau3AI	GATCCAACGTGTAAGTCTG-NH2

Supplementary Table 2: 1899-bp-long sequence of human HMGA2 CDS

1899-bp-long sequence of human HMGA2 CDS	GGAGTCTCCCCATCCTCCTTTGCTTTCCGACTGCCCAAGGCACTTTCAATCTCAATCT<br>CTTCTCTCTCTCTCTCTCTCTCTCTCTCTCTCTCTCTCTCTCTCTCTCTCTCTCTCGC<br>AGGGTGGGGGGAAGAGGAGGAGGAATTCTTTCCCCGCCTAACATTTCAAGGGACAC<br>AATTCACTCCAAGTCTCTTCCCTTTCCAAGCCGCTTCCGAAGTGCTCCCGGTGCCCG<br>CAACTCCTGATCCCAACCCGCGAGAGGAGCCTCTGCGACCTCAAAGCCTCTCTTCCT<br>TCTCCCTCGCTTCCCTCCTCCTCTTGCTACCTCCACCTCCACCGCCACCTCCACCTCC<br>GGCACCCACCCACCGCCGCCGCCGCCACCGGCAGCGCCTCCTCCTCTCCTCCTCCTC<br>CTCCCCTCTTCTCTTTTTGGCAGCCGCTGGACGTCCGGTGTTGATGGTGGCAGCGGC<br>GGCAGCCTAAGCAACAGCAGCCCTCGCAGCCCGCCAGCTCGCGCTCGCCCCGCCGG<br>CGTCCCCAGCCCTATCACCTCATCTCCCGAAAGGTGCTGGGCAGCTCCGGGGCGGTC<br>GAGGCGAAGCGGCTGCAGCGGCGGTAGCGGCGGCGGGAGGCAGGATGAGCGCACG<br>CGGTGAGGGCGCGGGGCAGCCGTCCACTTCAGCCCAGGGACAACCTGCCGCCCCA<br>GCGCCTCAGAAGAGAGGACGCGGCCGCCCCAGGAAGCAGCAGCAAGAACCAACC<br>GGTGAGCCCTCTCCTAAGAGACCCAGGGGAAGACCCAAAGGCAGCAAAAACAAGA<br>GTCCCTCTAAAGCAGCTCAAAAGAAAGCAGAAGCCACTGGAGAAAAACGGCCAAG<br>AGGCAGACCTAGGAAATGGCCACAACAAGTTGTTCAGAAGAAGCCTGCTCAGGAG<br>GAAACTGAAGAGACATCCTCACAAGAGTCTGCCGAAGAGGACTAGGGGGCGCCAA<br>CGTTCGATTTCTACCTCAGCAGCAGTTGGATCTTTTGAAGGGAGAAGACACTGCAGT<br>GACCACTTATTCTGTATTGCCATGGTCTTTCCACTTTCATCTGGGGTGGGGTGGGGTG<br>GGGTGGGGGAGGGGGGGTGGGGTGGGGAGAAATCACATAACCTTAAAAAGGACT<br>ATATTAATCACCTTCTTTGTAATCCCTTCACAGTCCCAGGTTTAGTGAAAAACTGCTG<br>TAAACACAGGGGACACAGCTTAACAATGCAACTTTTAATTACTGTTTTCTTTTTTCTT<br>AACCTACTAATAGTTTGTTGATCTGATAAGCAAGAGTGGGCGGGTGAGAAAAACCG<br>AATTGGGTTTAGTCAATCACTGCACTGCATGCAAACAAGAAACGTGTCACACTTGTG<br>ACGTCGGGCATTCATATAGGAAGAACGCGGTGTGTAACACTGTGTACACCTCAAATA<br>CCACCCCAACCCACTCCCTGTAGTGAATCCTCTGTTTAGAACACCAAAGATAAGGAC<br>TAGATACTACTTTCTCTTTTTCGTATAATCTTGTAGACACTTACTTGATGATTTTTAACT<br>TTTTATTTCTAAATGAGACGAAATGCTGATGTATCCTTTCATTCAGCTAACAAACTAG<br>AAAAGGTTATGTTCATTTTTCAAAAAGGGAAGTAAGCAAACAAATATTGCCAACTCT<br>TCTATTTATGGATATCACACATATCAGCAGGAGTAATAAATTTACTCACAGCACTTGTT<br>TTCAGGACAACACTTCATTTTCAGGAAATCTACTTCCTACAGAGCCAAAATGCCATTT<br>AGCAATAAATAACACTTGTCAGCCTCAGAGCATTTAAGGAAACTAGACAAGTAAAAT<br>TATCCTCTTTGTAATTTAATGAAAAGGTACAACAGAATAATGCATGATGAACTCACCT<br>AATTATGAGGTGGGAGGAGCGA

Supplementary Table 3: Original data of Figure 3. See Supplementary_Table_3

Supplementary Table 4: Sequences of real-time PCR primers

Gene Name	Forward	Reverse
HMGA2	5'-ACCCAGGGGAAGACCCAAA-3'	5'-CCTCTTGGCCGTTTTTCTCCA-3'
Bcl-2	5'-TTGCCAGCCGGAACCTATG-3'	5'-CGAAGGCGACCAGCAATGATA-3'
Caspase 3	5'- CATGGAAGCGAATCAATGGACT-3'	5'- CTGTACCAGACCGAGATGTCA-3'
GAPDH	5'-TGTGGGCATCAATGGATTTGG-3'	5'-ACACCATGTATTCCGGGTCAAT-3'
